# Magnetic Resonance Imaging Features of Progressive Condylar Resorption: A Case Report

**DOI:** 10.7759/cureus.36261

**Published:** 2023-03-16

**Authors:** Sonam Khurana, Mayank Pahadia, Pranav Parasher, Adriana G Creanga

**Affiliations:** 1 Oral and Maxillofacial Pathology, Radiology and Medicine, New York University College of Dentistry, New York, USA; 2 Oral and Maxillofacial Diagnostic Sciences, University of Florida, Gainesville, USA; 3 Diagnostic Radiology, Rutgers School of Dental Medicine, Newark, USA; 4 Oral and Maxillofacial Radiology, Rutgers School of Dental Medicine, Newark, USA

**Keywords:** mandibular condyle, degenerative joint disease, temporo mandibular joint, mri imaging, cone-beam computed tomography (cbct)

## Abstract

Progressive condylar resorption is a dysfunctional remodeling of the temporomandibular joint of unknown origin. It usually manifests in young girls and causes reduced ramus height, loss of condylar volume, steep mandibular angle, limited jaw motion, and pain. On magnetic resonance imaging, the condition is associated with anterior disc displacement with or without reduction. This article discusses imaging features of progressive condylar resorption that cause severe temporomandibular joint degenerative changes, emphasizing the careful evaluation of imaging changes of the temporomandibular joint in young female patients. The early diagnosis of progressive condylar resorption helps to reduce the further progression of the condition.

## Introduction

Progressive condylar resorption (PCR) of the temporomandibular joint (TMJ) is a dysfunctional remodeling of unknown origin [1]. The term “dysfunctions remodeling” is used if it adversely affects joint function and occlusion, whereas functional remodeling causes morphological changes without affecting joint functions and occlusion [2]. PCR is a noninflammatory disorder of the TMJ that is localized and causes degenerative changes, such as erosion and subsequent healing of the articular fibrocartilage and subchondral bone [3]. The etiology of the condition is unknown; that is why it is called "idiopathic condylar resorption" (ICR) [4]. The condition is also referred to as idiopathic condyles, condylar atrophy, aggressive condylar resorption, and acquired condylar hypoplasia [5]. Different authors have described PCR case reports and conducted studies to describe morphological changes in the condyle. The PCR usually occurs around puberty in females, and the incidence decreases after age 20 [3,6]. Imaging assessment plays a crucial role in determining the extent and treatment planning. In addition to clinical evaluation, imaging helps to assess the stability of PCR. Cone Beam Computed Tomography (CBCT) helps to evaluate bony changes in three dimensions more efficiently than conventional radiographs [7]. Magnetic resonance imaging (MRI) helps to evaluate soft tissue changes associated with degenerative changes due to PCR [3]. A few cases and studies are reported in the literature that describe PCR imaging features assessed through CBCT and MRI. CBCT is the standard of care in dental and ENT offices to assess bony changes. Our case report is rare because, in addition to CBCT, we report MRI features of PCR. We aim to emphasize the importance of soft tissue changes in addition to bony changes to complement the treatment planning. 

## Case presentation

**Figure 1:**
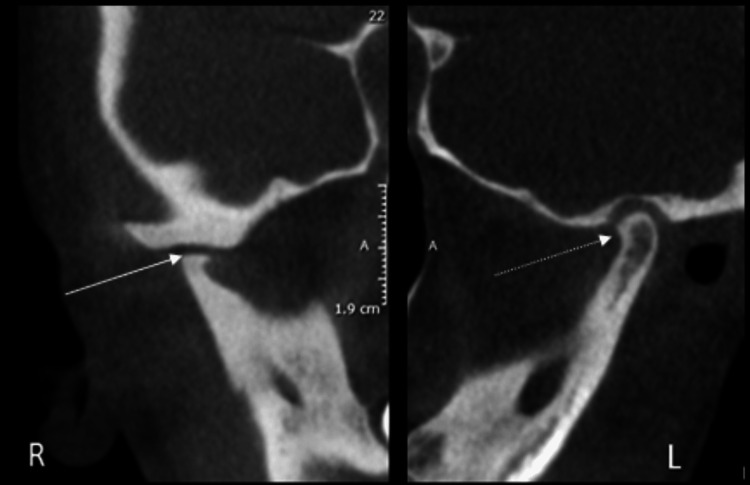
CBCT corrected sagittal sections in maximum intercuspation position showing right and left TMJ with severe degenerative changes on the right side (solid white arrow) and moderate flattening on the left side (dashed white arrow) CBCT: Cone Beam Computed Tomography, TMJ: temporomandibular joint

This is a case report of a 16-year-old female patient referred to Rutgers School of Dental Medicine. The patient was experiencing intermittent TMJ pain for the last four to five months on mouth opening. On CBCT evaluation, bilateral TMJ degenerative changes were noted. The right condyle was smaller in size and shape due to superior, anterior, and posterior aspect volume loss and severe flattening. A small osteophyte formation was also noted at the anterior aspect. The right articular eminence was severely flattened and reduced in size and shape (Figure 1). The condylar head was positioned slightly posteriorly within the glenoid fossa at the maximum intercuspation position.

**Figure 2:**
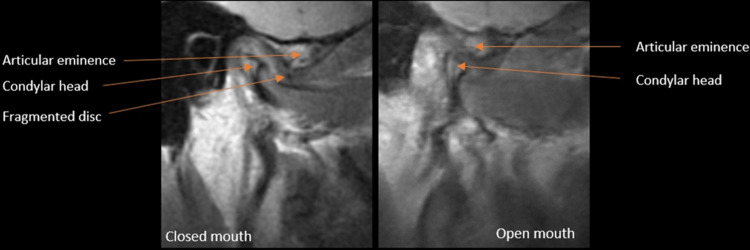
MRI Proton density showing fragmented disc on right side closed mouth view and no translation on the open mouth view

On sagittal MRI, proton density (PD), right side closed mouth position, the disc was fragmented and lost its typical bow-tie shape. In the open position, the condylar head showed slight rotation and no translation within the glenoid fossa. The disc was not reduced to its normal position because it was fragmented. On the T2 weighted images (T2WI), there was no fluid collection within the joint. No changes were noted in the lateral pterygoid muscle (Figure 2).

**Figure 3:**
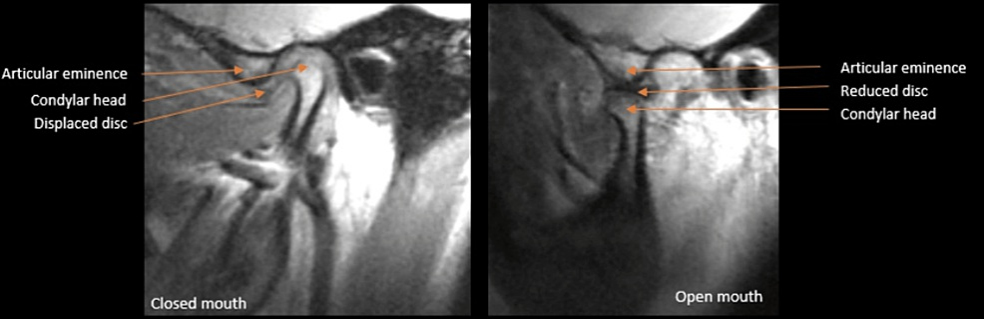
MRI Proton density showing anterior displaced disc on left side closed mouth view and reduced disc over condylar head on open mouth view

On the left side, the anterior and superior aspect of the condylar head was moderately flattened, with a slight notching at the anterior part. The cortices of the left condylar head, articular eminence, and glenoid fossa were thick, sclerotic, and continuous. The articular eminence was flattened and reduced in size and shape (Figure 1). The condylar head was centered within the glenoid fossa at the maximum intercuspation position. On sagittal MRI, PD showed a flattened disc; the bone marrow signal of the condylar head was normal. In the closed position, the posterior band was located anteriorly. In the open position, the head was situated immediately inferior to the crest of the eminence, suggesting a slightly limited anterior excursion; the disc was present immediately superior to the head of the condyle, reduced to its normal position (Figure 3).

**Figure 4:**
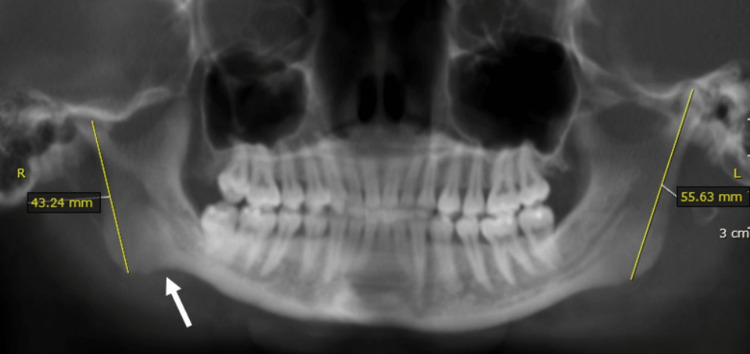
Panoramic reconstruction at the maximum intercuspation position showing decreased ramus length on the right side measured from Condylion to Gonion (indicated by measurements on each side) and deep antigonial notch (indicated by white arrow)

**Figure 5:**
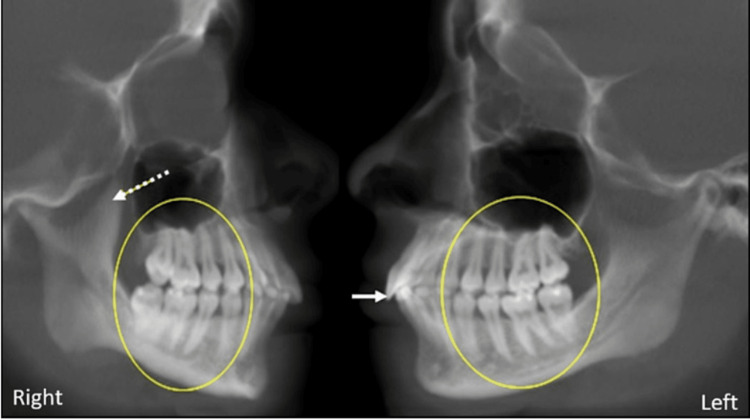
Corrected CBCT sagittal sections at the maximum inter-cuspation position (depicted by yellow circle), Right coronoid hyperplasia (depicted by white dotted arrow), Proclined anterior maxillary teeth (depicted by white solid arrow) CBCT: Cone Beam Computed Tomography

On the T2 WI, there was no fluid collection within the joint. There were no changes noted in the lateral pterygoid muscle. Other features noted were decreased ramus length measured from Condylion to Gonion on the right side, deviation of the mandible on the right side, deep antigonial notch (Figure 4), elongated right coronoid process, and proclined anterior maxillary teeth (Figure 5).

## Discussion

This case report describes a case of PCR with imaging emphasis. The findings in our case were like those mentioned in the literature [3]. Arnett and colleagues suggest two possible mechanisms for the pathophysiology of this condition. The first reason includes increased consistent forces on the joint, and the second includes decreased adaptive capacity of the articulating surfaces of the TMJ [1,2].

The disease usually affects bilaterally, but no symmetrical pattern exists. Unilateral involvement reduces the growth of the involved condyle and the mandible. It manifests as a decrease in the vertical dimension of the condyle, ascending ramus, and body of the mandible elevated occlusal plane and skeletal midline shift on the affected side. Maxilla may follow some mandibular changes, and the cranial base (fossa) may be depressed on the involved side. Bilateral involvement shows a dolichofacial growth pattern that may be associated with short condylar processes, short ramus, and mandibular body, and an increased vertical dimension of the anterior region of the mandible along with the labiolingual reduction in the dimensions of the alveolar process. The mandibular plane may be steep, and the gonial angles are obtuse. A clockwise facial growth pattern and an anterior open bite development are common findings. All the above changes can reduce airway dimensions secondary to small mandibular growth and posterior-inferior repositioning of the mandibular symphysis [3]. 

PCR causes severe osseous and occlusal changes due to its occurrence during the growth period. Imaging assessment plays a crucial role in determining the extent and treatment planning. In addition to clinical evaluation, imaging helps to assess the stability of PCR. CBCT helps to evaluate bony changes in three dimensions more efficiently than conventional radiographs [8]. MRI is the gold standard for evaluating soft tissue changes. Our case report is one of the rare to mention both CBCT and MRI changes. The case showed progressive bony changes on the right side along with anterior disc displacement without reduction. The PCR was still active, and disc perforation cannot be ruled without arthroscopy. On the left side, PCR was stable, with moderate bony changes and anterior disc displacement with reduction. The disc perforation is unlikely on the left side.

The differential diagnoses of PCR are juvenile rheumatoid arthritis (JRA) and degenerative joint disease. Juvenile rheumatoid arthritis is an autoimmune disorder that occurs at a younger age and includes other joints. Like JAR, PCR occurs in younger age groups; however, it does not involve other joints. Degenerative joint disease has similar radiographic features, and unlike PCR, it does not occur at a younger age [3].

The realistic approach to assess the PCR stability without any validated method includes bone scanning like technetium-99m methylenediphosphonate (99mTc-MDP) standard bone scans [9,10]. Bone scans are not specific to determine PCR stability. The other option includes reevaluation and comparison of anatomy after particular periods. The most important tool to evaluate stability is “time.” As evaluated in our case, assessing the anatomical changes and the extent through CBCT or computed tomography is advisable. If the disease is in its end stage, wait six to 12 months for radiographic reevaluation. Imaging stability exists if size, shape, quality, and spatial relationship remain constant without any change over time [3].

## Conclusions

PCR is a rapidly progressing disease entity that needs awareness among the dental community to improve their understanding of the condition. PCR has heterogeneous clinical and imaging features, which sometimes are very subtle to be noted on a conventional radiograph. The author highly recommends correlating clinical features or recent changes in occlusion with radiographs to formulate a better diagnosis.
